# Deriving effective vaccine allocation strategies for pandemic influenza: Comparison of an agent-based simulation and a compartmental model

**DOI:** 10.1371/journal.pone.0172261

**Published:** 2017-02-21

**Authors:** Özden O. Dalgıç, Osman Y. Özaltın, William A. Ciccotelli, Fatih S. Erenay

**Affiliations:** 1 Department of Management Sciences, University of Waterloo, Waterloo, Ontario, Canada; 2 Edward P. Fitts Department of Industrial and Systems Engineering, North Carolina State University, Raleigh, North Carolina, United States of America; 3 Department of Pathology and Molecular Medicine, McMaster University, Hamilton, Ontario, Canada; 4 Grand River Hospital, Kitchener, Ontario, Canada; Columbia University, UNITED STATES

## Abstract

Individuals are prioritized based on their risk profiles when allocating limited vaccine stocks during an influenza pandemic. Computationally expensive but realistic agent-based simulations and fast but stylized compartmental models are typically used to derive effective vaccine allocation strategies. A detailed comparison of these two approaches, however, is often omitted. We derive age-specific vaccine allocation strategies to mitigate a pandemic influenza outbreak in Seattle by applying derivative-free optimization to an agent-based simulation and also to a compartmental model. We compare the strategies derived by these two approaches under various infection aggressiveness and vaccine coverage scenarios. We observe that both approaches primarily vaccinate school children, however they may allocate the remaining vaccines in different ways. The vaccine allocation strategies derived by using the agent-based simulation are associated with up to 70% decrease in total cost and 34% reduction in the number of infections compared to the strategies derived by using the compartmental model. Nevertheless, the latter approach may still be competitive for very low and/or very high infection aggressiveness. Our results provide insights about potential differences between the vaccine allocation strategies derived by using agent-based simulations and those derived by using compartmental models.

## Introduction

Influenza is a highly contagious viral disease. Each year 5–15% of the world population is infected with influenza resulting in 3–5 million severe cases and 250,000–500,000 deaths [1]. Together with pneumonia, influenza is the seventh leading cause of death in the U.S. [2]. Considering the cost of medical treatments and working day losses, the annual burden of influenza epidemics to the U.S. economy was estimated as $87.1 billion in 2003 [3].

An emerging virus that spreads globally may lead to an influenza pandemic [4]. Unlike seasonal epidemics, pandemics occur abruptly and cause horrendous death tolls, e.g., the 1918 Spanish influenza pandemic infected around 500 million and killed approximately 50 million people worldwide [5]. Among pandemic mitigation interventions (e.g., social distancing, public health measures, antiviral prophylaxis), vaccination provides the most efficient and durable response [6, 7]. However, the supply of influenza vaccine is often scarce during a pandemic due to production limitations. Thus, the population is prioritized based on risk-factors related to influenza exposure and transmissibility when distributing the available doses [8].

In the literature, compartmental models [9–16] and agent-based simulations [17–22] are frequently employed to make mitigation plans for influenza pandemics and evaluate the effectiveness of various public health interventions [23–25]. Although it is known that the infection propagation is different in these two approaches [26], a detailed comparison of the strategies derived by using them is often omitted. On the one hand, compartmental models represent the number of individuals in each stage (or compartment) of the epidemic (e.g., susceptible, exposed, infected, recovered) by continuous-time state variables, and formulate the transitions among different compartments using differential equations. These models can rapidly evaluate many scenarios and intervention strategies, but they assume that individuals in each compartment mix uniformly and randomly with each other. Moreover, deterministic compartmental models do not consider the uncertainties in disease propagation (e.g., stochasticity in transmission events, incubation, and recovery periods) [22]. Thus, such models may not accurately model infection dynamics, especially at the initial and final stages of a pandemic when few infectious individuals exist [27]. Despite their simplifying assumptions, compartmental models have proved to be predictive [28–30], and they have been successfully extended to capture large-scale host heterogeneities. These extensions of the simple compartmental framework include age-specific contact patterns [13] and heterogeneities induced by spatial structure [31]. Agent-based simulations, on the other hand, consider uncertainties about the infection parameters, and they store individual-level information to model contact patterns in a population at the expense of increased computational burden [32–34].

Our goal in this paper is to identify possible scenarios under which performances of the effective age-specific vaccine allocation strategies derived by using compartmental models and agent-based simulations may differ significantly in practical settings. For this purpose, we consider an influenza pandemic in Seattle using a custom-built deterministic compartmental model and an agent-based simulation developed by Chao et al. (2010) [6]. The compartmental model is calibrated to closely approximate the results of the agent-based simulation under no vaccination. We apply mesh-adaptive optimization to derive effective age-specific vaccine allocation strategies based on four different objective functions. At each iteration of the optimization process, the performances of the newly generated vaccine allocation strategies are evaluated using the agent-based simulation in one set of experiments, and using the compartmental model in the other set of experiments. We perform sensitivity analysis to identify the differences between these two approaches under various vaccine coverage and infection aggressiveness scenarios.

We observe that the age-specific vaccine allocation strategies derived by using computationally expensive but more realistic agent-based simulation and those derived by using fast but more stylized compartmental model are different, although both models are calibrated to generate similar results under no vaccination. We use the agent-based simulation to evaluate the performances of strategies derived by the compartmental model. Our results show that the vaccine allocation strategies derived by the agent-based simulation are associated with up to 70% decrease in total cost and 34% reduction in the number of infections compared to the strategies derived by the compartmental model. Nevertheless, the latter approach may still be competitive for very low and/or very high infection aggressiveness scenarios.

It is clear that any two infectious disease spread models can differ from each other with respect to the assumptions in their design and parametrization. Furthermore, the degree to which each modeling approach allows for inclusion of heterogeneity and uncertainty varies. Therefore, the empirical comparison presented in this study is valid for the considered agent-based influenza pandemic simulation which is well-known and commonly used in the literature [6]. Our results, however, still provide important insights into the possible differences between the vaccine allocation strategies derived by using agent-based simulations and deterministic compartmental models.

## Materials and methods

We consider different levels of vaccine coverage and infection aggressiveness, and apply mesh-adaptive optimization [35] to find effective strategies for allocating limited vaccine doses to different age-groups in the population with respect to four performance measures: total cost, number of deaths, number of infections, and years of life lost. We evaluate the performances of the trial vaccine allocation strategies at each iteration of the optimization algorithm using an agent-based simulation in one set of experiments, and a compartmental model in the other set of experiments.

### Agent-based simulation

We employ FluTe, an open-source and validated agent-based pandemic influenza simulation developed by Chao et al. (2010) [6]. FluTe’s contact network is composed of census tracts divided into communities of 500–3,000 individuals. Each community consists of randomly generated households of 1–7 individuals in one of the five age-groups: preschool children (0–4), school children (5–18), young adults (19–29), adults (30–64), and seniors (65 and over). Individuals can be members of multiple community-based mixing groups such as households, household clusters (composed of socially close households), neighborhoods and communities. The simulation has two time epochs for each day: day- and night-time. At night time, individuals can only make contacts within their community-based mixing groups, whereas they may contact other individuals in day-time if they share the same social mixing group, e.g., daycare, school, workplace.

In each time epoch, a contact for potential disease transmission between any two individuals sharing a mixing group is generated. During a contact between a susceptible and an infectious individual, influenza transmission may occur with a probability that depends on vaccine efficacy, virus load and symptoms of the infectious individual. Each infected person follows a predefined daily viral load profile representing the level of infectiousness on each day of the disease duration. Infected individuals may become symptomatic after an asymptomatic incubation period of one to three days [36]. Symptomatic individuals are twice as infectious as the asymptomatic ones. Infected individuals recover and become immune after six days.

The vaccinated individuals have reduced likelihood of getting infected during a contact, becoming symptomatic when infected, and transmitting the disease [37]. The vaccine efficacy reaches its maximum level in two weeks with exponential increments, and the maximum vaccine efficacy varies among the age groups. Due to incremental nature of vaccine efficacy, the timing of vaccine interventions affects success in containing influenza pandemics. FluTe allows administrating vaccines before (pre-vaccination) or after (reactive vaccination) the onset of the pandemic. We refer the reader to Chao et al. (2010) for further details about FluTe [6].

### Compartmental model

We develop a deterministic compartmental model that closely approximates the results of FluTe for Seattle under no vaccination. Similar to FluTe, we divide the population into five age groups, *AG* = {preschool children (0–4), school children (5–18), young adults (19–29), adults (30–64), seniors (65+)}. Each age group *i* ∈ *AG* includes vaccinated and unvaccinated individuals in five compartments: susceptible (S), exposed (E), infected (I), recovered (R), and dead (D). We denote the susceptible individuals in age group *i* ∈ *AG* with vaccination status *h* ∈ *H* = {(u)nvaccinated, (v)accinated} by $S_{i}^{h}$, for example. The exposed compartment (E) corresponds to the asymptomatic individuals in FluTe. In addition, the infected compartment (I) corresponds to the symptomatic individuals in FluTe. A proportion of asymptomatic individuals never develop symptoms in FluTe, therefore, we split the exposed compartment into two sub-compartments: those who eventually show disease symptoms (*E*_→_*I*), and those who recover without showing symptoms (*E*_→_*R*). The symptomatic individuals are twice as infectious as asymptomatic ones in both FluTe and the SEIR model.

The incidence rate of new infections in age group *i* caused by infectious individuals in age group *j*, denoted by λ_*ij*_, is given by:

$$\lambda_{ij}=\frac{\Phi_{ij}\left(\beta_{j}^{u}\left(I_{j}^{u}+E_{j}^{u}/2\right)+\beta_{j}^{v}\left(I_{j}^{v}+E_{j}^{v}/2\right)\right)}{N_{j}}.$$ (1)

**Fig 1:**
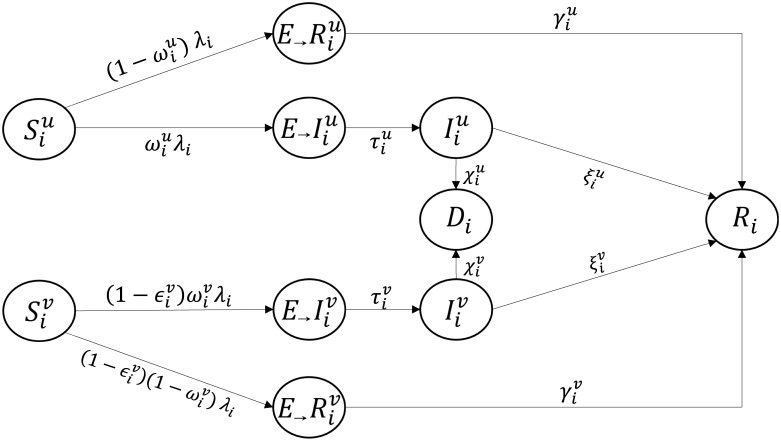
Transition rates between compartments.

In Eq (1), Φ_*ij*_ is the contact rate from age group *i* to *j*. Parameter $\beta_{j}^{u}$ ($\beta_{j}^{v}$) denotes the transmission rate of unvaccinated (vaccinated) infectious individuals in age group *j* given a single contact with a susceptible individual. Variables $I_{j}^{u}$ ($I_{j}^{v}$) and $E_{j}^{u}$ ($E_{j}^{v}$) represent the number of unvaccinated (vaccinated) infected and exposed individuals in age group *j*, respectively. Note that $E_{j}^{u}={\left(E_{\to }I\right)}_{j}^{u}+{\left(E_{\to }R\right)}_{j}^{u}$ and $E_{j}^{v}={\left(E_{\to }I\right)}_{j}^{v}+{\left(E_{\to }R\right)}_{j}^{v}$. Finally, *N*_*j*_ is the size of age group *j*, and ∑_*j*∈*AG*_ *N*_*j*_ = *N* where *N* denotes the total population size. The overall infection rate of individuals in age group *i* is equal to λ_*i*_ = ∑_*j*∈*AG*_ λ_*ij*_. Note that vaccinated (*v*) and unvaccinated (*u*) compartments are interdependent because the infection rate λ_*i*_ depends both on the number of vaccinated and unvaccinated infectious individuals. Fig 1 depicts the transitions among the compartments, and the model equations are given by:

$$\frac{dS_{i}^{h}}{dt}=-\left(1-\epsilon_{i}^{h}\right)\lambda_{i}S_{i}^{h}\;h\in H,i\in AG$$ (2a)

$$\frac{d{\left(E_{\to }I\right)}_{i}^{h}}{dt}=\left(1-\epsilon_{i}^{h}\right)\lambda_{i}\omega_{i}^{h}S_{i}^{h}-\tau_{i}^{h}{\left(E_{\to }I\right)}_{i}^{h}\;h\in H,i\in AG$$ (2b)

$$\frac{d{\left(E_{\to }R\right)}_{i}^{h}}{dt}=\left(1-\epsilon_{i}^{h}\right)\lambda_{i}\left(1-\omega_{i}^{h}\right)S_{i}^{h}-\gamma_{i}^{h}{\left(E_{\to }R\right)}_{i}^{h}\;h\in H,i\in AG$$ (2c)

$$\frac{dI_{i}^{h}}{dt}=\tau_{i}^{h}{\left(E_{\to }I\right)}_{i}^{h}-\left(\xi_{i}^{h}+\chi_{i}^{h}\right)I_{i}^{h}\;h\in H,i\in AG$$ (2d)

$$\frac{dR_{i}^{h}}{dt}=\xi_{i}^{h}I_{i}^{h}+\gamma_{i}^{h}{\left(E_{\to }R\right)}_{i}^{h}\;h\in H,i\in AG$$ (2e)

$$\frac{dD_{i}}{dt}=\sum_{h\in H}\chi_{i}^{h}I_{i}^{h}\;i\in AG$$ (2f)

Parameter $\epsilon_{i}^{h}\in \left[0,1\right]$ denotes the reduction in the likelihood of becoming infected after vaccination, naturally $\epsilon_{i}^{u}=0$ for unvaccinated individuals. Parameter $\omega_{i}^{h}\in \left[0,1\right]$ denotes the proportion of exposed individuals who eventually become symptomatic. Parameters $\tau_{i}^{h}$, $\gamma_{i}^{h}$, $\xi_{i}^{h}$, and $\chi_{i}^{h}$ denote the Exposed-to-Infected, Exposed-to-Recovered, Infected-to-Recovered, and Infected-to-Death transition rates, in that order. We set $\gamma_{i}^{h}=\frac{1}{1/\xi_{i}^{h}+1/\tau_{i}^{h}}$ to ensure that the exposed individuals who recover without showing symptoms stay asymptomatic during the course of the disease. The *D* compartment is included in the model for tracking the number of influenza-related deaths.

Let *p*_*i*_ denote the vaccinated proportion of age group *i*. Moreover, let *b*_*i*_ denote the initial number of infecteds in age group *i*. The boundary conditions ∀*i* ∈ *AG* are given by $S_{i}^{v}\left(0\right)=p_{i}\left(N_{i}-b_{i}\right)$, $S_{i}^{u}\left(0\right)=\left(1-p_{i}\right)\left(N_{i}-b_{i}\right)$, $I_{i}^{v}\left(0\right)=p_{i}b_{i}$, $I_{i}^{u}\left(0\right)=\left(1-p_{i}\right)b_{i}$, $E_{i}^{v}\left(0\right)=E_{i}^{u}\left(0\right)=R_{i}^{v}\left(0\right)=R_{i}^{u}\left(0\right)=D_{i}\left(0\right)=0$. We solve the system of differential Eq (2) numerically using the fourth-order Runge-Kutta method [38].

### Formulating the optimization problem

The optimization aims to find an effective age-specific allocation of a given vaccine supply *V* such that ∑_*i*∈*AG*_ *p*_*i*_*N*_*i*_ ≤ *V*. We consider four different performance measures:

- *Total expected cost* is equal to the sum of vaccination, infection and mortality costs. Vaccination cost ($c_{i}^{b}$) includes the vaccine price, work time lost, and the cost of potential side effects. Infection cost, which is different for unvaccinated ($c_{i}^{u}$) and vaccinated ($c_{i}^{v}$) individuals, refers to the sum of medication, outpatient visits and hospitalization expenses. Mortality cost ($c_{i}^{d}$) stands for the terminal care expenses.
- *Total number of infections* is equal to the number of individuals affected by the pandemic.
- *Total number of deaths* is equal to the number of influenza-related deaths.
- *Total years of life lost (YLL)* weighs each death with the expected remaining life time based on the U.S. life tables and the age distributions [39, 40].

In particular, at any time during the course of the pandemic, the four performance measures are calculated as follows:

$$\begin{array}{ll} \text{Total cost }\left(TC\right) & =\sum_{i\in AG}\left(c^{b}N_{i}p_{i}+c_{i}^{u}N_{I_{i}^{u}}+c_{i}^{v}N_{I_{i}^{v}}+c_{i}^{d}N_{D_{i}}\right),\\ \text{Total number of infections }\left(TI\right) & =\sum_{i\in AG}\left(N_{I_{i}^{u}}+N_{I_{i}^{v}}\right),\\ \text{Total number of deaths }\left(TD\right) & =\sum_{i\in AG}N_{D_{i}},\\ \text{Total YLL }\left(TY\right) & =\sum_{i\in AG}Y_{i}N_{D_{i}}, \end{array}$$

where *Y*_*i*_ denotes the YLL value of age group *i* ∈ *AG*. Moreover, $N_{I_{i}^{u}}$ ($N_{I_{i}^{v}}$), and $N_{D_{i}}$ represent the total number of infections among unvaccinated (vaccinated) individuals and influenza-related deaths in age group *i* ∈ *AG*, respectively.

### Calibration

We run FluTe using the population file for Seattle (around 560,000 residents) that is included in the software distribution package. Contact rates within mixing groups and infectious disease parameters of influenza are set based on the values estimated in Chao et al. (2010) so that attack rates are consistent with the 1957 Asian A(H2N2) and 2009 A(H1N1) influenza pandemics without vaccination [6]. We seed the model with 10 randomly generated infected people. The vaccinated individuals have 40% reduced probability of becoming infected, 40% reduced probability of becoming symptomatic given infection, and 67% reduced probability of transmitting infection. These values represent the effectiveness of a well-matched seasonal influenza vaccine [41]. The vaccine is only 60% as effective in seniors as everyone else, since older people with weaker immune systems often have a lower immune response to influenza vaccine [42].

The homogeneous mixing assumption of the compartmental model (referred to as SEIR model hereafter) results in faster and more diverse disease spread, whereas the infection follows a more tranquil pattern in FluTe, i.e., individuals can transmit the disease only to those in their contact list. For a fair comparison of the age-specific vaccine allocation strategies derived by using the SEIR model and FluTe, we calibrate the SEIR model so that the number of new infections for each day of the pandemic closely matches to the corresponding average outcome from FluTe over 100 replications under no vaccination. In particular, we calibrate the SEIR model by varying the contact rates (Φ_*ij*_), transmission rates ($\beta_{i}^{h}$), and initial number of infections (*b*_*i*_). As a goodness of fit measure, we use Pearson’s chi-square statistic (*χ*^2^ measure), and employ the numerical optimization algorithm described in the following section to find parameters of the SEIR model that minimize the maximum *χ*^2^ measure over all age groups.

**Fig 2:**
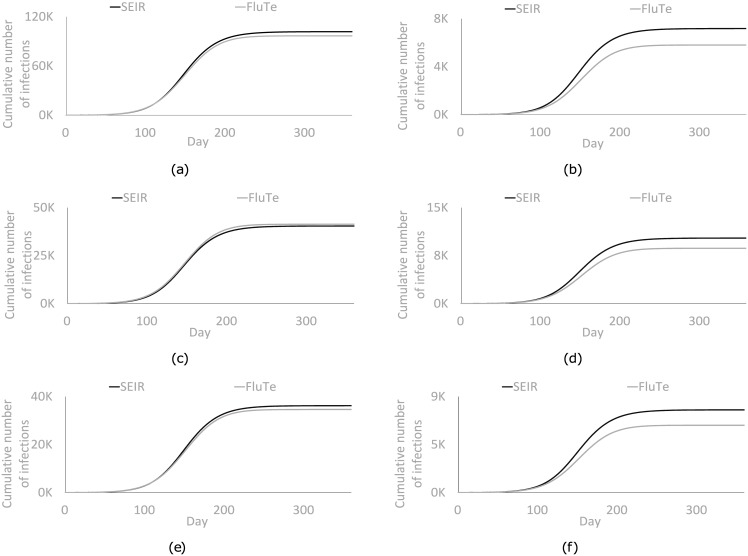
Cumulative number of infections in each age group of FluTe and the SEIR model after the calibration process for *R*_0_ = 1.2 without vaccination. (a) Total population. (b) Preschool children (0–4). (c) School children (5–18). (d) Young adults (19–29). (e) Adults (30–64). (f) Seniors (65+).

We first perform the calibration for *R*_0_ = 1.2. We then repeat the process for each *R*_0_ value considered in our numerical analyses using the previous calibration results as the initial solution. We keep the contact rates the same as those found for *R*_0_ = 1.2 (see S1 Table) because initial tests show that further calibration of the contact rates for different *R*_0_ values is not necessary to obtain good matches between the results of FluTe and the SEIR model. Fig 2a depicts the cumulative number of infections in each day after the calibration process. The cumulative number of infections in different age groups are presented in Fig 2b–2f. Furthermore, S2 Table in the supplement shows the similarity between the age-specific attack rates in FluTe and in the SEIR model under no vaccination. The parameters of FluTe and the calibrated SEIR model are provided in Table 1. We do not present the parameters of FluTe related to network structure and virus load profile and refer the readers to Chao et al. (2010) for more details [6].

**Table 1:** Input parameters of FluTe and the SEIR model.

	SEIR	FluTe				Source
Latent period duration (LPD)	1.9 days	1.9 days^‡^				[6]
Total disease duration (TDD)	6 days	6 days				[6]
Infectious period duration (IPD)	4.1 days	4.1 days				TDD−LPD
Transition rate						
Exposed-to-Infectious(*τ*^*u*^)	0.52632	–				(LPD)^−1^
Exposed-to-Recovered (*γ*)	0.1667	–				(TDD)^−1^
Infectious-to-Recovered (*ξ*)	0.2439	–				(IPD)^−1^
Likelihood						
of showing the symptoms (*ω*^*u*^)^†^	0.67	0.67				[6]
		Transmission rate/probability (*β*^*u*^)*		Initial number of infections (*b*)		
		SEIR	FluTe	SEIR	FluTe	
Basic reproduction number (*R*_0_)	1.2	0.0044	0.2057	19.48	10	Calibration and [6]
	1.4	0.0053	0.2435	22.07	10	
	1.6	0.0063	0.2812	7.88	10	
	1.8	0.0071	0.3190	5.01	10	
	2.0	0.0077	0.3567	5.01	10	
	2.2	0.0083	0.3944	5.01	10	
	2.4	0.0089	0.4322	5.01	10	
Age group specific disease parameters						
	0–4	5–18	19–29	30–64	65+	
Population size	36,722	124,787	71,601	265,047	65,303	[6]
Death rate (unvaccinated-*χ*^*u*^)	0.0049	0.002	0.0056	0.0038	0.001	[13]
Death rate (vaccinated-*χ*^*v*^)	0.0012	0.0005	0.0017	0.0011	0.0004	[13]
Vaccine efficacy in						
becoming infected	40%	40%	40%	40%	24%	[6]
transmitting the disease	40%	40%	40%	40%	24%	[6]
showing the symptoms	67%	67%	67%	67%	40%	[6]
Performance related parameters						
	0–4	5–18	19–29	30–64	65+	
Infection cost (unvaccinated)($)	275.3	275.3	328.98	328.98	492.56	[13]
Infection cost (vaccinated)($)	231.58	231.58	264.71	264.71	404.54	[13]
Terminal care cost($)	3,435	3,435	7,605	7,605	8,309	[13]
Vaccination cost ($)	37.26	37.26	37.26	37.26	37.26	[13]
YLL (years)	79.42	70.71	57.92	36.45	13.37	[39, 40]

^‡^: Mean of a discrete distribution of 1, 2, or 3 days with probabilities 0.3, 0.5, and 0.2.

^†^: These numbers are for the unvaccinated individuals. For the vaccinated individuals, they should be multiplied by (1- vaccine efficacy in showing the symptoms).

*: These numbers are for the unvaccinated individuals. For the vaccinated individuals, they should be multiplied by (1- vaccine efficacy in transmitting the disease).

### Solution approach

The pandemic propagation is nonlinear because the incidence of new infections depends both on the current number of infectious and susceptible individuals. Moreover, the size and duration of outbreaks are uncertain in agent-based simulations like FluTe. All of these factors render traditional gradient-based optimization methods inapplicable. We therefore use a derivative-free approach, in particular, the mesh-adaptive direct search (MADS) algorithm as implemented in open-source software NOMAD [43]. Starting from an initial solution, the MADS algorithm iteratively tries to improve the current best solution by generating trial points on a mesh, which is a discretization of the variable space. Each iteration is composed of two main steps: the search and the poll steps. The search step evaluates a number of trial mesh points. If an improved mesh point is found, then the next iteration is initiated with the new incumbent solution using a larger mesh size. Whenever the search step fails to generate an improved mesh point, then the poll step is invoked. The poll step explores the variable space near the current incumbent solution. If the poll step also fails to improve the current best solution, then the mesh size and poll size parameters are reduced in order to increase the search resolution. The MADS algorithm stops after a given number of iterations or when the mesh size reaches a precision limit. We refer the reader to Le Digabel (2011) for more information about the MADS algorithm and NOMAD [44].

All numerical experiments are conducted using a PC with 48 cores (2.85 Ghz and 128 GB memory). We run both FluTe and the SEIR model up to one year. We generate 10 trial solutions in each iteration of the MADS algorithm. In one set of experiments, we use FluTe to evaluate the performance of trial solutions (FluTe+MADS), and in another set of experiments we use the SEIR model (SEIR+MADS). When using FluTe, we perform 24 replications to estimate the average performance of each trial solution. Based on our initial experiments, this sample size is sufficient to reduce the effects of sample variance on the results. We terminate the MADS algorithm after 1,000 trial solutions or when the mesh size is less than or equal to 10^−13^. The SEIR model evaluates trial solutions much faster than FluTe. Therefore, when using the SEIR model, we terminate the MADS algorithm after 100,000 trial solutions or when the mesh size is less than or equal to 10^−13^. We select *p*_*i*_ = 0.5, ∀*i* ∈ *AG* as the initial solution in the MADS algorithm, i.e., vaccinate 50% of the population in each age group. Although this solution may be infeasible under some vaccine coverage scenarios, it is still a proper initial solution because the MADS algorithm allows constraint violations in the intermediate iterations to diversify the search.

## Results

We present the age-specific vaccine allocation strategies derived by FluTe+MADS and SEIR+MADS. We highlight the age groups prioritized by each approach and evaluate the relative performance of the proposed strategies under various scenarios for multiple objective functions.

The basic reproductive number (*R*_0_) represents the average number of infections generated by a typical infectious person in a completely susceptible population. If *R*_0_ > 1, the infection spreads in the population. Otherwise, the infection eventually dies out without any intervention [45]. Fraser et al. (2009) estimated the *R*_0_ value of the 2009 H1N1 pandemic between 1.4 and 1.6 [46]. Medlock and Galvani (2009) reported that 30% vaccine coverage can mitigate a pandemic like the 2009 H1N1 if there is no delay in response time (i.e., vaccination starts on the first day of the pandemic) [13]. We therefore use *R*_0_ = 1.6, 30% vaccine coverage, and no delay in response time as the base case in our experiments. Note that the result of Medlock and Galvani (2009) depends on vaccine efficacy. They assumed 80% vaccine efficacy against infection for 0 to 64-year-olds and 60% for those 65 and older. More than 30% coverage would be required for less effective vaccines (or a mismatched vaccine). Moreover, the whole process of producing a pandemic vaccine for a novel influenza virus takes four to six months [47]. Therefore, a base case scenario with no response delay may seem unreasonable. However, we consider no other pandemic interventions such as quarantine, public health measures and antivirals, which may slow down the pandemic spread substantially. This effect may render the no response delay scenario more acceptable as a base case. In addition, analyzing the no response delay scenario is important to evaluate the maximum potential of a particular vaccination policy and coverage level to mitigate the pandemic.

Table 2 reports the age-specific vaccine allocation strategies derived by FluTe+MADS and SEIR+MADS in the base case. SEIR+MADS vaccinates only school children (5–18) to minimize the total cost (TC) objective. For other objectives, preschool (0–4) and school children are mainly vaccinated, and the remaining vaccines are allocated to young adults (19–29). FluTe+MADS vaccinates school children for the most part, and allocates the remaining vaccines to preschool children and young adults. Observe that SEIR+MADS uses fewer vaccine doses than FluTe+MADS for the TC objective, possibly because the effect of vaccination is more pronounced in the SEIR model as a result of the homogeneous mixing assumption.

**Table 2:** Vaccine allocation strategies obtained by FluTe+MADS and SEIR+MADS under the base-case scenario.

Performance measure		Vaccination fractions for each age group				
		0–4	5–18	19–29	30–64	65+
TC	SEIR+MADS	-	87%	-	-	-
	FluTe+MADS	-	99%	34%	-	-
TI	SEIR+MADS	100%	100%	9%	-	-
	FluTe+MADS	89%	100%	9%	1%	1%
TD	SEIR+MADS	100%	100%	9%	-	-
	FluTe+MADS	5%	97%	50%	3%	2%
TY	SEIR+MADS	100%	100%	9%	-	-
	FluTe+MADS	21%	98%	50%	1%	-

*R*_0_ = 1.6, 30% vaccine coverage, no delay in response time. **TC:** Total cost, **TI:** Total number of infections, **TD:** Total number of deaths, **TY:** Total YLL

In S1 Fig, we compare the overall attack rates of the SEIR model and FluTe under the same vaccination policy: the available vaccine stocks are primarily allocated to school children, the remaining doses are first allocated to preschool children, and then to young adults. For each coverage level and *R*_0_ value, the SEIR model has resulted in less overall attack rate than Flute, illustrating the more pronounced effect of vaccination in the former model. Furthermore, the critical vaccination fraction to stop the epidemic is lower in the SEIR model, and the amount of vaccine used relative to this threshold affects the optimal allocation. For example, if one can stop an epidemic by vaccinating only school children, there is no reason to vaccinate others.

We evaluate performances of the vaccine allocation strategies derived by FluTe+MADS and SEIR+MADS using FluTe with 100 replications. As seen in Table 3, the strategy from FluTe+MADS is significantly better than the one from SEIR+MADS for the TC objective. This is mainly due to the fact that the amount of vaccine used in the strategy from SEIR+MADS, while containing the disease effectively in the SEIR model, is insufficient to do so in FluTe.

**Table 3:** Objective values of recommended vaccine allocation strategies.

Performance measure	Sample mean		Difference	p-value
	SEIR+MADS	FluTe+MADS		
TC ($M)	23.2	6.5	16.7	<0.001
TI (infections)	1,170.3	945.2	225.1	0.117
TD (deaths)	10.7	9.9	0.8	0.584
TY (life years lost)	459.8	367.5	92.3	0.094

*R*_0_ = 1.6, 30% vaccine coverage, no delay in response time. The performance measures are calculated using FluTe with 100 replications.

### Sensitivity analysis on R_0_

**Fig 3:**
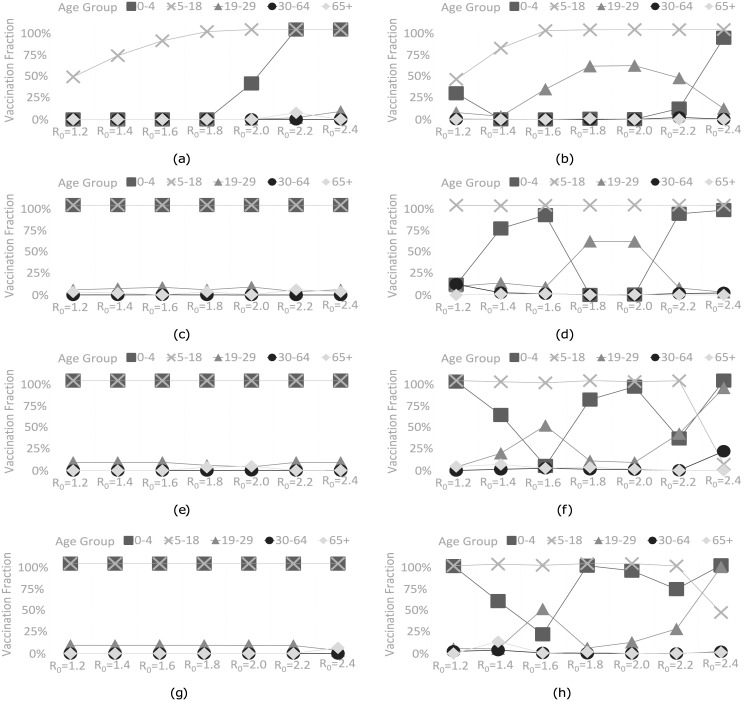
Vaccine allocation strategies derived by FluTe+MADS and SEIR+MADS under all objective functions for various R_0_ values (30% vaccine coverage, no delay in response time). (a) SEIR+MADS with the TC objective. (b) FluTe+MADS with the TC objective. (c) SEIR+MADS with the TI objective. (d) FluTe+MADS with the TI objective. (e) SEIR+MADS with the TD objective. (f) FluTe+MADS with the TD objective. (g) SEIR+MADS with the TY objective. (h) FluTe+MADS with the TY objective.

We vary the basic reproduction number *R*_0_ between 1.2 and 2.4 under 30% vaccine coverage with no delay in response time to analyze the sensitivity of the proposed vaccine allocation strategies to *R*_0_. For the total cost (TC) objective, SEIR+MADS increases the vaccinated proportion of school children from 47% to 100% as *R*_0_ increases from 1.2 to 2.0 (see Fig 3a). After covering school children, the remaining vaccines are allocated to preschool children when *R*_0_ ≥ 2.0. FluTe+MADS vaccinates 45% of school children as well as a small portion of preschool children and young adults when *R*_0_ = 1.2 (see Fig 3b). Furthermore, FluTe+MADS covers all school children when *R*_0_ ≥ 1.6. SEIR+MADS allocates 35% of the available vaccine when *R*_0_ = 1.2 and all of the available vaccine when *R*_0_ ≥ 2.2. On the other hand, FluTe+MADS allocates 43% of the available vaccine when *R*_0_ = 1.2 and all of the available vaccine when *R*_0_ ≥ 1.8. Intuitively, both methods use fewer vaccine doses for smaller *R*_0_ values to keep the vaccination cost low.

For the total number of infections (TI) objective, SEIR+MADS recommends a similar strategy for each *R*_0_; vaccinate all preschool and school children as well as small portions of young adults and/or seniors (65+) (see Fig 3c). However, vaccine allocation strategies from FluTe+MADS vary by *R*_0_ (see Fig 3d). These strategies cover all school children and allocate the remaining vaccines to preschool children and young adults when *R*_0_ ≥ 1.4. Note that both approaches use all available vaccine stocks, as vaccination cost is no longer a concern for the TI objective. This result is also valid for the total number of deaths (TD) and total YLL (TY) objectives.

For the TD and TY objectives, there is a trade off between reducing the number infections (by vaccinating school children) and reducing the casualties in high-mortality age groups (by vaccinating preschool children and young adults). SEIR+MADS again vaccinates all preschool and school children for all *R*_0_ values (see Fig 3e and 3g). FluTe+MADS covers all school children in addition to some proportions of preschool children and young adults when *R*_0_ ≤ 2.2 (see Fig 3f and 3h). However, when *R*_0_ = 2.4, FluTe+MADS vaccinates preschool children and young adults for the most part rather than school children. This unique case is due to high mortality rates of preschool children and young adults (see Table 1). The vaccine stocks become insufficient to contain the pandemic when *R*_0_ = 2.4, therefore, the recommended strategy focuses on minimizing casualties in high-mortality age groups.

The vaccine allocation strategies from FluTe+MADS perform better than those from SEIR+MADS when *R*_0_ is between 1.4 and 2.2, i.e., when the effective vaccine allocation becomes critical to control the pandemic (see S2 Fig). However, SEIR+MADS performs better than FluTe+MADS or the difference is not significant when *R*_0_ < 1.4 or *R*_0_ > 2.2. Recall that we run the MADS algorithm for at most 1,000 trial solutions in FluTe+MADS, while we allow for 100,000 trial solutions in SEIR+MADS. Therefore, it is not unlikely that SEIR+MADS finds better strategies than FluTe+MADS, especially when a small vaccination level is enough to contain the infection (i.e., *R*_0_ < 1.4). On the other hand, when the infection is very aggressive, i.e., *R*_0_ > 2.2, 30% vaccine coverage in the base case is not enough for containing the pandemic, and therefore, there is limited room for improvement by optimizing the vaccine allocation. One exception to this pattern is for the TD and TY objectives where FluTe+MADS performs 7% and 9% better than SEIR+MADS, respectively, by prioritizing high-mortality age groups, i.e., preschool children and young adults when *R*_0_ = 2.4.

### Sensitivity analysis on vaccine coverage and response time

We set *R*_0_ = 1.6 and analyze the sensitivity of the age-specific allocation strategies to vaccine coverage and response time. Table 4 presents the strategies obtained under 20%, 30% and 40% vaccine coverage with no response delay (i.e., vaccination starts on the first day of the pandemic). SEIR+MADS vaccinates preschool and school children for the most part, and allocates the remaining vaccines to young adults for all objectives except the total cost (TC) objective for which 87% of school children is vaccinated under all coverage levels to reduce the vaccination cost. FluTe+MADS vaccinates school children, and allocates the remaining vaccines to preschool children and young adults.

**Table 4:** Vaccine allocation strategies for different coverage scenarios.

		FluTe+MADS					SEIR+MADS				
	Vaccine coverage	Vaccination fraction for each age group (*p*_*i*_)					Vaccination fraction for each age group (*p*_*i*_)				
		0–4	5–18	19–29	30–64	65+	0–4	5–18	19–29	30–64	65+
**TC**	20%	1%	88%	1%	-	-	-	87%	-	-	-
	30%	-	99%	34%	-	-	-	87%	-	-	-
	40%	34%	98%	22%	2%	3%	-	87%	-	-	-
**TI**	20%	2%	87%	2%	-	1%	-	90%	-	-	-
	30%	89%	100%	9%	1%	1%	100%	100%	9%	-	-
	40%	64%	98%	99%	2%	1%	100%	100%	84%	-	3%
**TD**	20%	15%	80%	-	-	10%	-	90%	-	-	-
	30%	5%	97%	50%	3%	2%	100%	100%	9%	-	-
	40%	6%	100%	96%	11%	-	100%	100%	80%	2%	-
**TY**	20%	34%	70%	6%	-	12%	-	90%	-	-	-
	30%	21%	98%	50%	1%	-	100%	100%	9%	-	-
	40%	64%	98%	98%	2%	2%	84%	100%	95%	-	-

*R*_0_ = 1.6, no response delay

The strategies from FluTe+MADS outperforms SEIR+MADS in all objective types when vaccine coverage is 30% (see S3 Fig). However, strategies from SEIR+MADS for the total number of deaths (TD) and the total YLL (TY) objectives on average perform better than those derived by FluTe+MADS when the vaccine coverage is 20% or 40% (see S3 Fig). If the vaccine coverage is 20%, there is a small set of effective vaccine allocation strategies that can contain the pandemic. On the other hand, if the vaccine coverage is 40%, the set of effective strategies is large with many local optimums. In both cases, a large number of trial solutions should be evaluated. FluTe+MADS may not find an effective strategy with 1,000 trial solutions when the vaccine coverage is 20%, and gets stuck at a local optimum when the vaccine coverage is 40%. Furthermore, the random noise around the objective values of each FluTe replication may make it difficult for the MADS algorithm to find an effective improvement direction.

Table 5 presents the allocation strategies for different response time scenarios with *R*_0_ = 1.6 and 30% vaccine coverage. Prevaccination refers to the case where the vaccine is administered two weeks before the beginning of the pandemic so that it reaches its maximum effectiveness by the time the virus starts spreading. Others refer to the scenarios where the vaccination starts *d* days after the onset of the outbreak (*d* = 0, 10, 20, 30, 40, 60, 80, or 90). In these delayed response scenarios, the vaccine effectiveness will gradually increase and reach its maximum level in two weeks during the course of the pandemic both in the SEIR model and FluTe [6].

**Table 5:** Vaccine allocation strategies for different response time scenarios.

		SEIR+MADS					FluTe+MADS				
	Response time (days)	Vaccination fraction for each age group (*p*_*i*_)					Vaccination fraction for each age group (*p*_*i*_)				
		0–4	5–18	19–29	30–64	65+	0–4	5–18	19–29	30–64	65+
**TC**	P	–	86%	–	–	–	19%	100%	–	–	4%
	0	–	87%	–	–	–	–	99%	34%	–	–
	10	–	89%	–	–	–	77%	100%	15%	–	5%
	20	–	91%	–	–	–	46%	100%	31%	–	4%
	40	–	96%	–	–	–	–	96%	49%	5%	–
	60	–	100%	–	–	–	23%	83%	71%	–	8%
	80	1%	100%	–	–	–	48%	6%	–	54%	–
	90	72%	100%	–	–	–	4%	5%	–	55%	–
**TI**	P	100%	100%	1%	–	9%	16%	100%	43%	–	–
	0	100%	100%	9%	–	–	89%	100%	9%	1%	1%
	10	100%	100%	9%	–	–	67%	99%	16%	2%	2%
	20	100%	100%	7%	1%	–	–	100%	39%	5%	–
	40	100%	100%	9%	–	–	19%	100%	–	14%	–
	60	100%	100%	9%	–	–	30%	93%	52%	–	5%
	80	100%	100%	1%	2%	–	56%	65%	88%	–	4%
	90	100%	100%	1%	2%	–	59%	40%	–	36%	–
**TD**	P	100%	100%	9%	–	–	13%	100%	17%	9%	1%
	0	100%	100%	9%	–	–	5%	97%	50%	3%	2%
	10	100%	100%	9%	–	–	–	100%	60%	–	–
	20	100%	100%	9%	–	–	14%	100%	27%	6%	6%
	40	100%	100%	9%	–	–	–	100%	60%	–	–
	60	100%	100%	5%	1%	–	66%	98%	23%	–	8%
	80	100%	100%	9%	–	–	100%	–	100%	22%	–
	90	100%	100%	9%	–	–	87%	–	100%	24%	–
**TY**	P	100%	100%	9%	–	–	71%	100%	10%	2%	6%
	0	100%	100%	9%	–	–	21%	98%	50%	1%	–
	10	100%	100%	9%	–	–	93%	98%	–	4%	–
	20	100%	100%	9%	–	–	36%	100%	37%	–	4%
	40	100%	100%	9%	–	–	1%	100%	59%	–	–
	60	100%	100%	9%	–	–	100%	100%	9%	–	1%
	80	100%	100%	9%	–	–	100%	47%	100%	–	–
	90	100%	100%	9%	–	–	100%	100%	–	2%	–

*R*_0_ = 1.6, 30% vaccine coverage. **P**: Prevaccination.

In Table 5, SEIR+MADS vaccinates preschool children and school children for all objectives except for the TC objective. More interestingly, the recommended allocation strategy is rather insensitive to response delay for the TI, TD and TY objectives. This result is due to the fact that the impact of vaccination is so pronounced in the SEIR model that the infection can still be contained by vaccinating high transmitters even after 90-day response delay.

The vaccine allocation strategies from FluTe+MADS, however, vary significantly with response time, although vaccinating the school children is still the main focus in most cases. For the total number of deaths (TD) objective, FluTe+MADS increases the vaccinated proportion of age groups who have high mortality risk as the response is further delayed. When the response delay is 80 days, for example, the vaccinated proportion of school children drops to 0% and young adults are fully covered. This result complies with the literature which suggests prioritizing vaccination of high transmitters (e.g., school children) earlier and prioritizing vaccination of those who have high mortality rate (e.g., young adults, preschool children) later in the pandemic [14, 48, 49].

The strategies from FluTe+MADS generally outperform those from SEIR+MADS for different response time scenarios (see S3 Fig). The performance difference mainly decreases as the response is further delayed, except for the TD objective, where significant differences are obtained by focusing on high-risk age groups even when the response delay is more than 60 days.

## Conclusions

Health policy makers commonly use agent-based simulations and compartmental models when evaluating and designing effective vaccine allocation strategies. Our study shines a light on the possible differences between the strategies obtained by these two approaches. In particular, we observe that age-specific vaccine allocation strategies derived by using a computationally expensive but realistic agent-based simulation and those derived by using a fast but stylized compartmental model may be different from each other. They, however, both recommend vaccinating school children for the most part, which complies with the literature [19, 49].

The age-specific vaccine allocation strategies derived by using the agent-based simulation significantly outperforms those derived using the compartmental model especially for moderate levels of basic reproduction number (i.e., 1.4 ≤ *R*_0_ ≤ 2.2) when vaccine stocks are not very scarce. In other cases, either it is rather easy to control the pandemic (e.g., when *R*_0_ = 1.2), so there are several strategies that can effectively control the infection, or vaccination is not sufficient to control the pandemic. In such extreme scenarios, the performance gap between the two approaches is small because there is limited room for improvement.

We also note the following two observations. First, SEIR+MADS tries to limit the number of infected individuals for all *R*_0_ levels using almost the same vaccine allocation strategy for all objective functions under all scenarios. On the other hand, strategies from FluTe+MADS vary significantly for different objective functions and parameter scenarios seeking a balance between the number of infections and influenza-related deaths. Second, the impact of vaccination is very significant in SEIR+MADS possibly due to the homogeneous mixing assumption. Therefore, once evaluated by FluTe, the performance of the strategies from SEIR+MADS appears to be less effective compared those derived by FluTe+MADS. Similar observations are reported by other studies that evaluate the performances of strategies derived by compartmental models using agent-based simulations [20].

Our observations summarized above are based on the comparison of a specific agent-based simulation and a compartmental model. However, FluTe is commonly used and well-received in the literature [50–52]. Therefore, although they are not directly generalizable, our observations are likely to hold for other agent-based simulations and compartmental models whose assumptions are similar to those analyzed in this study. The differences between the age-specific vaccine allocation strategies derived by SEIR+MADS and FluTe+MADS are possibly due to the combined effect of considering the heterogeneity in contact patterns and stochasticity in disease progression. However, we left measuring the individual effect of heterogeneity and stochasticity for future research as such an analysis requires a more simplified methodological setting.

There are a few limitations of our analysis. First, we examine age-specific vaccine allocation but do not consider other important risk factors, e.g., chronic medical conditions and pregnancy. Moreover, we use a deterministic numerical optimization algorithm, which does not incorporate the variance in the simulation replications when updating the search direction. Using a faster agent-based simulation, the number of replications can be increased to reduce the variance. Alternatively, ranking and selection methods can be applied to find the proper number of replications for each vaccine allocation strategy, which is left for future research.

## Supporting information

S1 Table The daily contact rate of age group i and age group j (Φ_ij_) used in the SEIR model after the calibration. We assume that the total number of daily contacts between age groups *i* and *j* is symmetric. To calculate the daily contact rate of someone in age group *i* with age group *j*, we divided the total number of daily contacts between *i* and *j* by the population of age group *i*. So, the age-specific contact rates are asymmetric because the population of age groups *i* and *j* are different.
(PDF)

S2 Table Age specific influenza attack rates in Asian A(H2N2) pandemic and simulations of metropolitan Seattle using FluTe and the SEIR model for R_0_ = 1.6 without vaccination. (PDF)

S1 Fig Overall attack rates for different vaccine coverage levels and R_0_ values. (a) FluTe. (b) SEIR model. Both Flute and the SEIR model evaluate the same vaccine allocation policy for all vaccine coverage levels. In this policy, available vaccine stocks are primarily allocated to school children, the remaining doses are first allocated to preschool children, and then to young adults.
(EPS)

S2 Fig Objective values of vaccine allocation strategies for different R_0_ values (30% vaccine coverage, no delay in response time). (a) Total cost. (b) Number of infections. (c) Mortality. (d) Years of life lost.
(EPS)

S3 Fig Objective values of vaccine allocation strategies for different coverage scenarios (R_0_ = 1.6, no delay in response time) and response time scenarios (R_0_ = 1.6, 30% vaccine coverage). (a) Total cost. (b) Number of infections. (c) Number of deaths. (d) Years of life lost.
(EPS)
