# Supplementary material for: Deriving effective vaccine allocation strategies for pandemic influenza: Comparison of an agent-based simulation and a compartmental model
Source: PLoS One. 2017 Feb 21;12(2):e0172261. doi: 10.1371/journal.pone.0172261 (PMC5319753; doi:10.1371/journal.pone.0172261)
Supplement: S1 Table — We assume that the total number of daily contacts between age groups i and j is symmetric. To calculate the daily contact rate of someone in age group i with age group j, we divided the total number of daily contacts between i and j by the population of age group i. So, the age-specific contact rates are asymmetric because the population of age groups i and j are different. (PDF) [file pone.0172261.s001.pdf]

i \ j	**0-4**	**5-18**	**19-29**	**30-64**	**65+**
**0-4**	2.98	32.69	4.71	0.20	0.62
**5-18**	9.62	35.49	13.36	48.31	8.44
**19-29**	2.41	23.28	1.26	0.55	0.40
**30-64**	0.03	22.75	0.15	1.56	2.43
**65+**	0.35	16.13	0.43	9.85	0.48
