# Supplementary material for: Deriving effective vaccine allocation strategies for pandemic influenza: Comparison of an agent-based simulation and a compartmental model
Source: PLoS One. 2017 Feb 21;12(2):e0172261. doi: 10.1371/journal.pone.0172261 (PMC5319753; doi:10.1371/journal.pone.0172261)
Supplement: S2 Table — (PDF) [file pone.0172261.s002.pdf]

**Age Group**	FluTe [1]	SEIR	Asian A (H2N2) [2]
Preshool children	38%	39%	35%
School children	53%	56%	55%
Young adults	26%	30%	25%
Adults	28%	29%	20%
Seniors	23%	26%	14%
**Overall**	33%	35%	31%

References

[1] Chao DL, Halloran ME, Obenchain VJ, Longini Jr IM. FluTE, a publicly available stochastic influenza epidemic simulation model. PLoS Computational Biology. 2010;6(1):e1000656.

[2] Longini IM, Ackerman E, Elveback LR. An optimization model for influenza A epidemics. Mathematical Biosciences. 1978;38(1-2):141–157.
